# Terpyridine and Quaterpyridine Complexes as Sensitizers for Photovoltaic Applications

**DOI:** 10.3390/ma9030137

**Published:** 2016-02-27

**Authors:** Davide Saccone, Claudio Magistris, Nadia Barbero, Pierluigi Quagliotto, Claudia Barolo, Guido Viscardi

**Affiliations:** Department of Chemistry and NIS Interdepartmental Centre, University of Torino, Via Giuria 7, I-10125 Torino, Italy; davide.saccone@unito.it (S.D.); claudio.magistris@unito.it (C.M.); nadia.barbero@unito.it (N.B.); pierluigi.quagliotto@unito.it (P.Q.); guido.viscardi@unito.it (G.V.)

**Keywords:** dye-sensitized solar cells, polypyridines, Ru(II) complexes, terpyridines, quaterpyridines

## Abstract

Terpyridine and quaterpyridine-based complexes allow wide light harvesting of the solar spectrum. Terpyridines, with respect to bipyridines, allow for achieving metal-complexes with lower band gaps in the metal-to-ligand transition (MLCT), thus providing a better absorption at lower energy wavelengths resulting in an enhancement of the solar light-harvesting ability. Despite the wider absorption of the first tricarboxylate terpyridyl ligand-based complex, Black Dye (BD), dye-sensitized solar cell (DSC) performances are lower if compared with N719 or other optimized bipyridine-based complexes. To further improve BD performances several modifications have been carried out in recent years affecting each component of the complexes: terpyridines have been replaced by quaterpyridines; other metals were used instead of ruthenium, and thiocyanates have been replaced by different pinchers in order to achieve cyclometalated or heteroleptic complexes. The review provides a summary on design strategies, main synthetic routes, optical and photovoltaic properties of terpyridine and quaterpyridine ligands applied to photovoltaic, and focuses on n-type DSCs.

## 1. Introduction

Dye-sensitized solar cells (DSCs) are photoelectrochemical devices able to convert sunlight into electricity [1]. The architecture and operating principles of these devices have already been extensively reviewed in the literature [2,3,4,5,6], and the photosensitizer represents one of the key components of this device. Different kinds of sensitizers [3,4] have been used so far, including Ru complexes [7], porphyrines [5], phtalocyanines, metal-free dyes [6] (including squaraines [8,9,10], cyanines [11,12], and push-pull dyes [13]).

**Figure 1:**
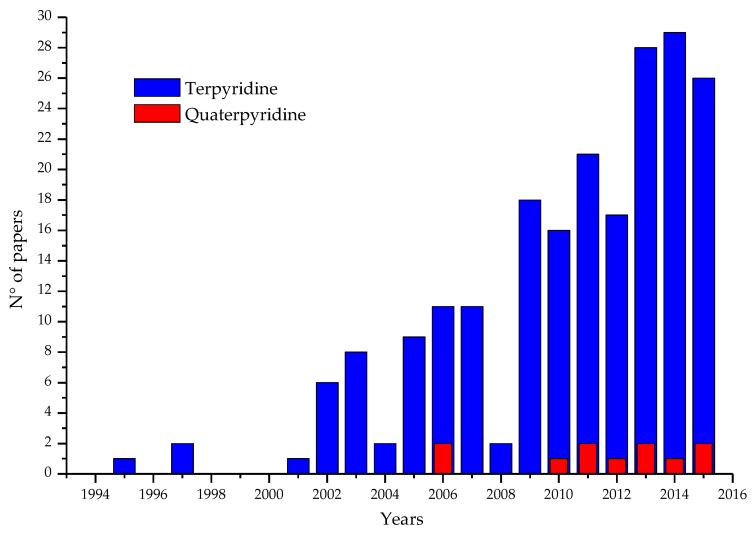
Publications concerning the use of terpyridines (**blue**) and quaterpyridines (**red**) in DSCs. Source: SciFinder (January 2016) [15].

Since 1997 [14] the interest in 2,2’:6’,2’’-terpyridine (tpy) as ligands in organometallic sensitizers for DSC applications has constantly grown and, in the last three years, more than 80 papers and patents concerning this subject were published. Interest on 2,2’:6’,2’’:6’’,2’’’-quaterpyridines (qtpy) is more recent and has resulted in more than 10 papers (Figure 1).

While the general use of polypyridines in Ru complexes sensitizers has already been deeply reviewed in the past by Islam [16], Vougioukalakis [17], and Adeloye [18], or for the electrolytes by Bignozzi *et al.* [19], no insight about the specific structure–properties relationships of tpy and qtpy complexes in the same field have been provided. Thus, we drew our attention on these panchromatic sensitizers with a particular focus on cells performances and device investigation. For this reason works dealing only with computational investigation [20] will not be taken into consideration.

**Figure 2:**
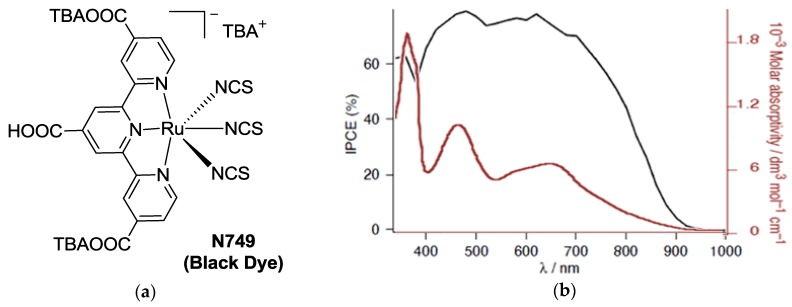

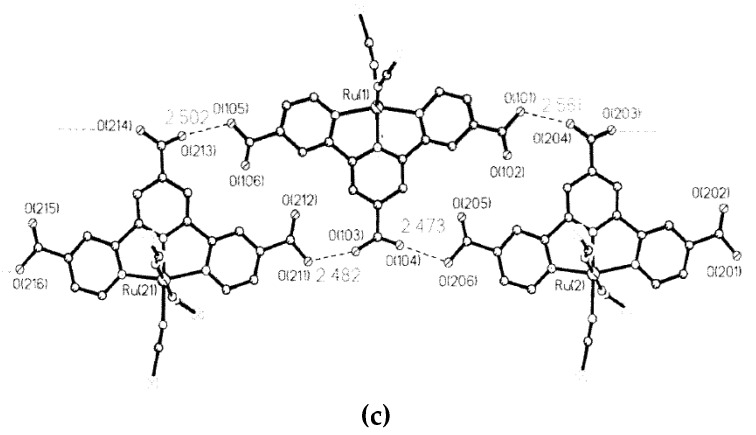
(**a**) Black Dye (BD) or N749 structure; (**b**) light absorption spectrum (**red**) and IPCE (**black**) [12] (Adapted from Ref 12 with permission of The Royal Society of Chemistry); and (**c**) crystal structure showing intermolecular hydrogen bonding [21] (Reprinted with permission from Nazeeruddin, M. K.; Péchy, P.; Renouard, T.; Zakeeruddin, S. M.; Humphry-Baker, R.; Comte, P.; Liska, P.; Cevey, L.; Costa, E.; Shklover, V.; Spiccia, L.; Deacon, G. B.; Bignozzi, C. A.; Grätzel, M. Engineering of efficient panchromatic sensitizers for nanocrystalline TiO_2_-based solar cells. J. Am. Chem. Soc. 2001, 123, 1613–1624. Copyright 2001 American Chemical Society).

The first use of tpy ligands in DSCs technology was pioneered by Nazeeruddin *et al.* [14], providing good performances owing to their broader absorption with respect to the standard bipyridine-based Ru complexes. The structure proposed in 1997 by the EPFL researchers was named N749 or Black Dye (BD), thanks to its panchromatic absorption (Figure 2, top) and represents a benchmark standard as tpy complex sensitizer. In this dye, ruthenium(II) is complexed by a tpy, the 4,4’,4’’-tricarboxy-2,2’:6’,2’-terpyridine (tctpy) and three isothiocyanate ancillary ligands. X-ray diffraction showed a slightly distorted octahedral coordination around the Ru atoms by the three nitrogen donors of tctpy and three nitrogen of isothiocyanate ligands. Very strong intermolecular bonds account for bidimensional arrays, in which the distance between the planes prevents π-stacking between the tpy rings (Figure 2, bottom) [21]. The final BD was prepared by titration with tetrabutylammonium hydroxide in order to deprotonate two of the three carboxylic functions, which proved to be a crucial feature for performances’ optimization. 

**Figure 3:**
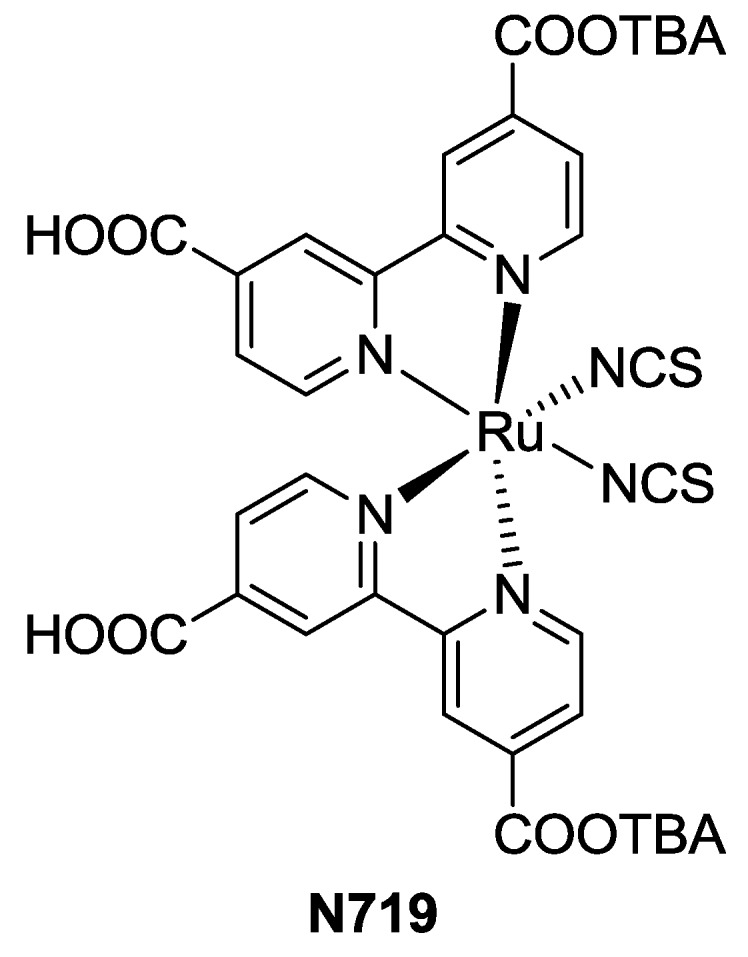
N719 structure.

Comparing to bipyridine structures, terpyridines allow to achieve lower band gap for the metal to ligand transition (MLCT), thus providing a better absorption at lower energies and, therefore, broader solar harvesting. The conversion efficiency of BD was first reported as 10.4% (TiO_2_: 18 μm, dye: 0.2 mM ethanol + 20 mM sodium taurodeoxycholate, electrolyte: 0.6 M DMPII (1,2-dimethyl-3-propylimidazolium iodide), 0.1 M I_2_, 0.5 M t-bupy (*t*-butylpyridine), 0.1 M LiI in methoxyacetonitrile) [21], and after further structural tuning (see Section 3.2.5), it was improved up to 11.2% (TiO_2_: 15 + 7 μm; dye 0.3 mM ethanol / *t*-butanol 1:1 with 0.6 mM of tetra-butylammonium deoxycholate and 1 mM deoxycholic acid (DCA) as co-adsorbate; electrolyte: 0.6 M DMPII, 0.05 M I_2_, 0.5 M t-bupy, 0.1 M LiI, 0.1 M GuNCS (guanidinium thiocyanate) in CH_3_CN) [22]. Despite the wider absorption, performances of BD are not superior to N719 [23] (Figure 3) or other optimized bipyridines complexes [24]. This behavior has been attributed to a lower molar extinction coefficient (7640 M^−1^·cm^−1^ in DMF) [21] and worse surface coverage of titania [25].

With the aim to further improve BD performance, several modifications have been carried out concerning each component of the complex. In order to increase the molar extinction coefficient and other features ruthenium was substituted with other metals; thiocyanates were replaced with different pinchers in order to obtain cyclometalated or heteroleptic complexes; and the terpyridine ligand was substituted with a quatertpyridine in order to extend the π-conjugation. 

The state of the art of polypyridine structures designed to further improve BD performances is summarized in the next sections. After a survey on the synthetic pathways to obtain tpy and qtpy structures, the three main types of changes underlined before (metal centre, ancillary, and tpy ligands) and their effect on DSCs performances will be taken into account in order to outline a structure-property relationship. Moreover, we remind that DSCs are a complex multivariate system [26], with different components and variables, and that a direct correlation between the photosensitizers’ molecular structures and related efficiencies can sometimes lead to inaccurate conclusions. For this reason, we selected literature examples where an internal standard reference (BD, 719 or N3) is reported in order to compare the characteristics of the novel structures. Moreover, specific conditions have been added to selected references.

## 2. Synthesis

The terpyridine structure was first synthesized in 1932 by Morgan and Burstall [27] as a byproduct of bipyridine synthesis, obtained by dehydrogenation of pyridine in the presence of anhydrous ferric chloride. Nowadays, several synthetic pathways have been developed [28,29,30], allowing this ligand to reach large applications such as uses in the preparation of Co(II) [31], Os(II) [32], Ru(II) [33] Ir(II) [34,35], Pd(II), Pt(II), and Au(III) complexes [36], supramolecular complexes [37,38,39,40], molecular wires [41], polymers [42], in the surface functionalization of nanostructures [43], in the conjugation with amino acids [44], biomacromolecules [45], in the coupling with inorganic nanoparticles [46], and have shown their remarkable activity in other fields such as sensing [47] and catalysis [48,49]. We will report briefly the main strategies used to obtain tpy ligands focusing on the structure–properties relationship in DSCs.

### 2.1. Terpyridine Core

**Scheme 1:**
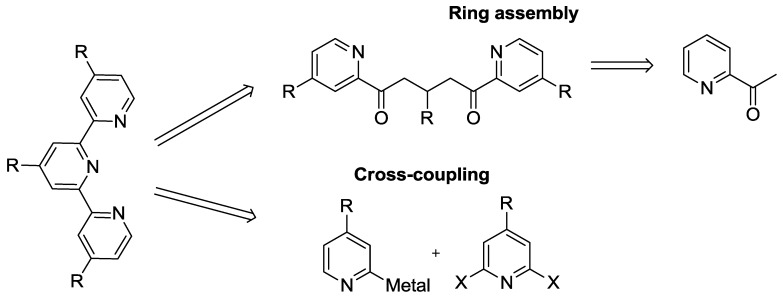
Retrosynthetic pathways to tpy core.

Tpy structures are mainly prepared through two basic synthetic approaches, which involve either ring assembly or coupling methodologies, as summarized in Scheme 1. 

**Scheme 2:**
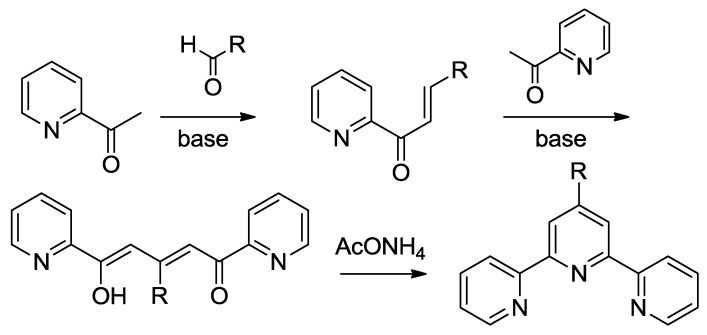
Example of the Kröhnke pathway.

The first route has been formerly reviewed in 1976 by Kröhnke [50], who reported the synthesis of α,β-unsaturated ketones from 2-acetyl derivatives of pyridine and aldehydes. Then, the intermediate reacts with another 2-acetylpyridine to form a 1,5-diketone that can undergo cyclization to pyridine thanks to ammonia sources such as AcONH_4_ (Scheme 2). A series of modifications to this procedure has been proposed in order to increase yields or improve the synthetic pathway sustainability [28,51].

The second strategy exploits recent advances in organometallic reactions (cross-coupling in Scheme 1). The electron poor pyridines are less effective in the Suzuki reaction [52] due to the weaker electrophilicity of pyridyl-boronates with respect to other organometallic reagents, such as the organo-tin involved in Stille reaction [53].

Noteworthy, the synthetic pathway used to achieve 4,4’,4’’-tricarboxy-2’,6’-terpyridine (tctpy) for Black Dye [54] involves the formation of the terpyridine core starting from 4-ethyl pyridine refluxed with Pd/C over nine days. This procedure was further improved by Dehaudt *et al.* [55]. Among the other possible strategies to obtain a tpy core, it is worth noting an inverse Diels-Alder reaction on 1,2,4-triazine that uses 2,5-norbornadiene as dienophile [56].

### 2.2. Functionalization of Terpyridines

In order to design complexes suitable for DSCs applications a series of modifications has to be taken into consideration, with the aim of introducing anchoring moieties, donor groups, bulky alkyl chains, or extending the π-conjugation. Cross-coupling reactions represent the most frequently used synthetic tool, while more specific pathways include the formation of carboxylic acid by furan degradation [57,58,59,60]. Other common syntheses are dealing with pyridine functionalizations; for example, the pyridine N-oxide is used as an intermediate to obtain halogen and pyrrolidinyl functionalizations [61,62], while 4-pyridones analogues are used to have access to halogens or triflates derivatives [63]. Husson *et al.* reviewed the derivatizations with thienyl [56] and furanyl [64] moieties while recently Woodward *et al.* [65] reported a synthetic strategy to further extend the scope and number of the anchoring moieties on oligopyridines.

### 2.3. Quaterpyridine Synthesis and Complex Formation

The synthesis and functionalization of qtpy usually exploit the same synthetic strategies used for tpy, namely Kröhnke and coupling reactions. In the latter case N-methyliminodiacetic acid (MIDA [66]) boronates have been successfully applied as key reagents to obtain quaterpyridine ligands in good yields [67] through Suzuki-Miyaura reaction.

**Scheme 3:**
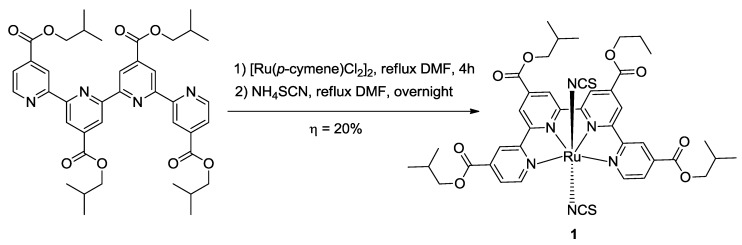
Microwave-assisted synthesis of the trans-Ru (II) complex [68].

In order to obtain Ru(II) complexes of polypyridines, Adeloye *et al.* [18] used Ru *p*-cymene or Ru(III)Cl_3_ as starting materials and they substituted the chlorines with thiocyanates or other ancillary ligands. Exploiting microwave-assisted synthesis, a facile procedure to obtain a functionalized qtpy ligand and its *trans*-dithiocyanato ruthenium complex has been reported [68] (Scheme 3). 

## 3. Modifications of Black Dye and Structure-Properties Relationships on Devices

### 3.1. Terpyridine modification

In this section, tpy based ruthenium complexes bearing three thiocyanates as ancillary ligands will be reviewed, outlining structural modifications on tpy ligand and their effects on DSCs performances.

Molecular engineering on tpy ligands has commonly the aim to extend π-conjugation in order to increase the molar extinction coefficient and further stabilize the LUMO level. In this way more photons can be harnessed and converted thanks to a simultaneous hyperchromic effect and bathochromic shift in the absorption spectra, respectively. Other common structural modifications are the substitution of one of the three pyridines with either a donor group (such as triphenyl amine), in order to enhance the push-pull system character, or a hydrophobic group, in order to reduce recombination with the electrolyte. Particularly interesting are the structural variations related to the anchoring moieties. The tctpy used in BD offers three possible anchoring points, allowing a proper sensitizer-semiconductor coupling and improving the stability of the device. Moreover, alternative anchoring groups, with respect to the carboxylic acid functionality, have been tested. Zakeeruddin [25] proposed a terpyridine functionalized with a phosphonic acid group on 4’-position with the purpose of overcoming the slow desorption of the carboxyl anchoring group from the semiconductor surface in presence of water. Waser [69] proposed a tpy bearing a phosphonic acid functionality, coupled with TiO_2_ for DSCs and water splitting applications, while Anthonysamy *et al.* [70] proposed a 4’-methacryloyloxymethylphenyl moiety as an anchoring group.

**Figure 4:**
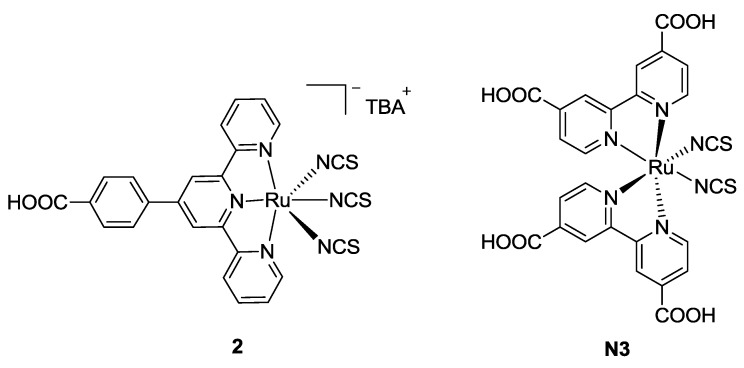
Structure proposed by Wang *et al.* and N3 dye [71].

As far as the carboxyl anchoring group is concerned, in 2002 Wang *et al.* [71] tested a 4’-carboxyphenyl substitution (Figure 4), obtaining an appreciable bathochromic shift with respect to N3 (*cis*-diisothiocyanato-bis(2,2’-bipyridyl-4,4’-dicarboxylic acid) ruthenium(II)), but a sensible loss in short circuit current in comparison with BD occurred, which can be explained by the fewer grafting points on the structure.

**Figure 5:**
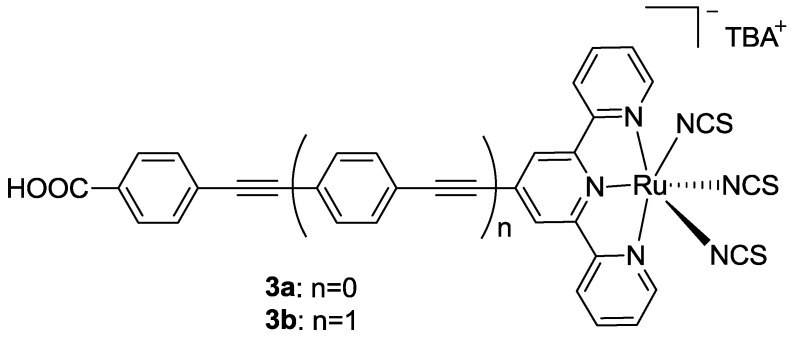
Complexes reported by Funaki *et al.* [72].

Funaki *et al.* [72] proposed a similar substitution, in which phenylene ethylene moieties (**3a** in Figure 5) were introduced between the COOH functionality and the tpy core, obtaining a better charge injection (12.8 mA/cm^2^) with respect to dye **2** (6.1 mA/cm^2^), even if a thicker TiO_2_ (36 μm *vs.* 10 μm) and higher light intensity (100 mW/cm^-2^ *vs.* 78 mW/cm^-2^) were used. The injection efficiency proved to be lower with respect to BD (16.7 mA/cm^2^), tested in the same conditions. Moreover, when the spacer was represented by two phenylene ethynylene units (**3b** in Figure 4) a higher molar extinction coefficient and slight bathochromic shift were obtained, but a significantly lower *J_sc_* value was observed (5.7 mA/cm^2^) which was ascribed to an increased dye aggregation.

McNamara *et al.* [73] reported a ligand similar to **2** bearing a hydroxamic acid instead of the carboxyl moiety. The dye showed promising properties but was not tested on any device. 

**Figure 6:**
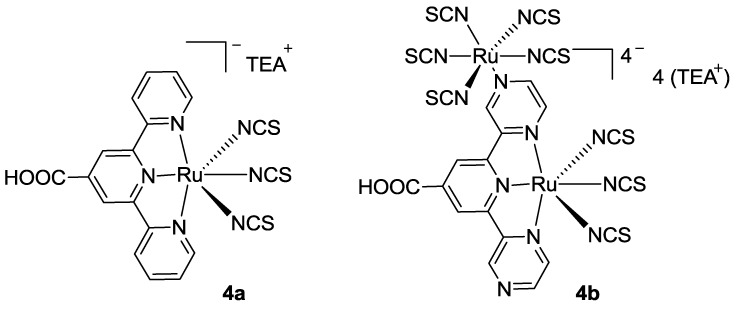
Complexes with one (**4a**) or two (**4b**) metal centers [74].

In 2010, Vougioukalakis *et al.* [74] synthesized a 4’-carboxyterpyridine acid Ru(II) complex (**4a** in Figure 5). With the purpose of increasing the chelating sites, the two outer pyridine rings were also substituted with pyrazine, which resulted in the coordination of a second Ru(II) atom (**4b** in Figure 6).

The overall performances were worse with respect to BD, even if a better absorption on TiO_2_ was recorded, due to the greater flexibility of the dyes bearing only one anchoring group, which accounts for a higher number of molecules adsorbed on the surface. Complex **4a**, whose structure is similar to dye **2**, showed similar *J_sc_* (6.19 mA/cm^2^), but its absorption was hypsochromically shifted with respect to BD. The 2,6-dipyrazinylpyridine ligand (complex **4b**) led to overall lowest performances with 0.27 mA/cm^2^ charge injection and 0.02% efficiency (TiO_2_: 22 μm, dye 0.3 mM ethanol, electrolyte PMII Ionic Salt, Dyesol). Further improvements in the number of chelated Ru(II) atoms have been reported by Manriquez *et al.* [75] in the preparation of supramolecular structures.

**Figure 7:**
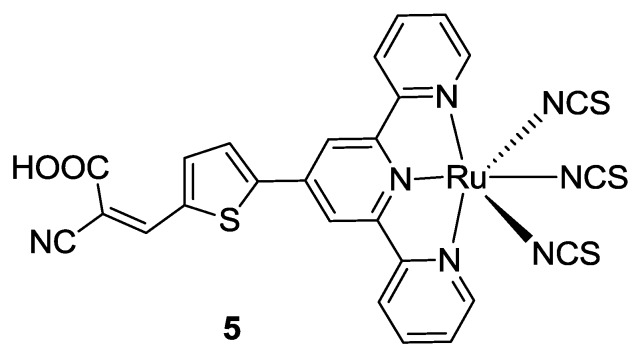
Terpyridine with a cyanoacrylic acid moiety [76].

Very recently, Kaniyambatti [76] reported a tpy substituted in 4’- with a cyanoacrylic acid moiety via a thiophene bridge (**5** in Figure 7). The modification leads again to a hypsochromic shift in the absorption spectrum coupled with a higher molar extinction coefficient owing to the extended π-conjugation and strong auxochrome resulting from the thiophene moiety.

**Figure 8:**
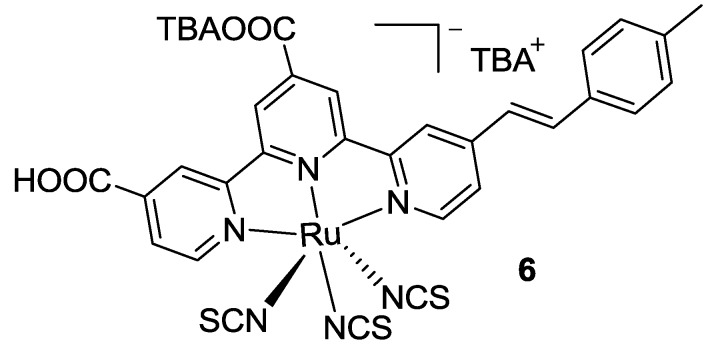
4-Methylstyryl substituted and double-anchored tpy (HIS-2) [77].

In 2013, Numata *et al.* [77] proposed a double anchored tpy bearing a 4-methylstyryl substituted in 4’’-position (**6** in Figure 8) in order to extend the π-conjugation and to obtain better charge injection with respect to N749. This complex achieved a higher molar extinction coefficient especially on the π-π* transition, and a better IPCE in the same region, which led to an improved efficiency with respect to BD (*η* = 11.1% ; TiO_2_: 25 μm; dye: 0.3 mM acetonitrile / *t*-butanol 1:1, 24 h + 20 mM CDCA, electrolyte: 0.05 mM I_2_, 0.1 M LiI, DMPII, 0.2 M t-bupy in CH_3_CN).

**Figure 9:**
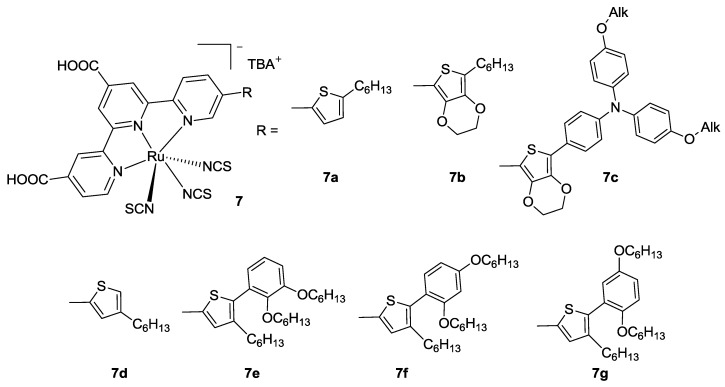
Series of 5’’-substituted tpy proposed by Yang (**7a-c**) [78]; and Kimura (**7d-g**) [79].

In 2011 Yang *et al.* [78] tested a series of 4,4'-dicarboxy terpyridine bearing a thiophene or a 3,4-ethylenedioxythiophene in 5’’ position (**7a,b** in Figure 9). The substitution of the latter with a triphenylamino moiety (**7c**) resulted in better performances with respect to BD tested in the same conditions (*η* = 8.29% *vs.* 6.89%; TiO_2_: 10 μm + 5 μm, dye: 0.3 mM ethanol + 10 mM chenodeoxycholic acid (CDCA), electrolyte: 0.6 M MDPII, 0.5 M t-bupy, 0.05 M I_2_, 0.1 M LiI in CH_3_CN), owing to the higher molar extinction coefficients in the high energy region of the spectrum. Substitution with hexyl-EDOT (**7b**, EDOT: 3,4-ethylenedioxythiophene) afforded even higher efficiency (*η* = 10.3% with TiO_2_: 15 + 5 μm). Similar modifications have been taken into consideration by Kimura *et al.* [79] (**7d–g** in Figure 9). In the series, structures with hindered hexyloxy-substituted rings resulted in better performances, probably because of the hindrance of alkyl chains towards the electrolyte, thus avoiding the redox couple to interact with titania and considerably reducing the dark current. Among these, the best results were obtained when the electron donor hexyloxy groups on the phenyl ring are in ortho or para positions (**7f** in Figure 9).

**Figure 10:**
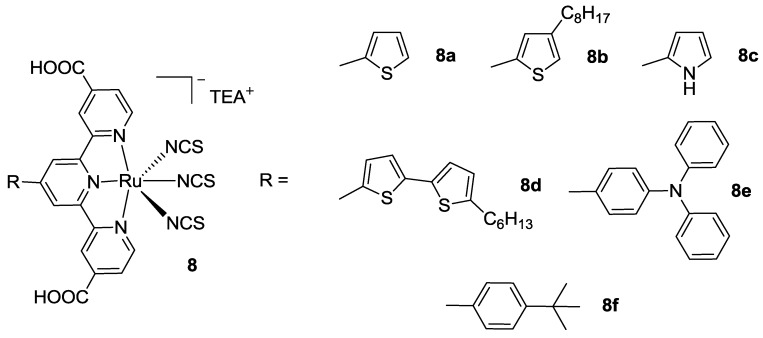
4’ substituted Black Dye analogs [80].

Very recently, Dehaudt [80] and Koyyada [81] proposed a simple synthetic pathway to achieve 4’-substituted Black Dye analogs (Figure 10) using octylthiophene (**8b**) and hexyl bithiophene (**8d**), pyrrole (**8c**), triphenylamine (**8e**), *t*-butyl phenyl (**8f**), phenoxazine, and phenothiazine groups. While these modifications did not allow to achieve better results respect to the BD in terms of efficiency, they gave an insight into the structure-property relationships, as well as fundamental issues about charge transfer, polarization, or binding. Thienyl-substituted analogues showed better performances with respect to triphenylamino donors, giving an efficiency of 5.57% (TiO_2_: 14 + 3 μm, dye: 0.5 mM ethanol / *t*-butanol + 10 mM CDCA, electrolyte: 0.5 M DMPII, 0.5 M t-bupy, 0.1 M LiI, 0.05 M I_2_ in CH_3_CN).

**Figure 11:**
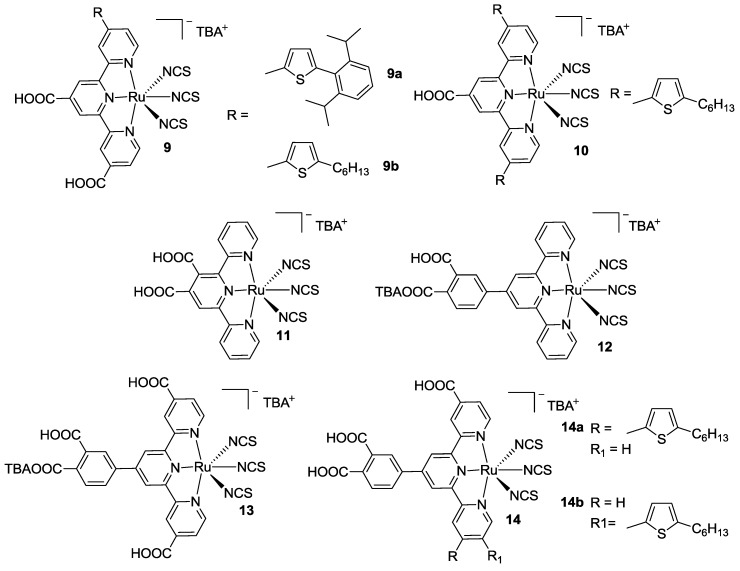
Structures proposed by Ozawa *et al.* [82,83,84,85,86,87].

Ozawa *et al.* proposed a series of tpy having anchoring groups either in the classical 4-, 4’- and 4’’-positions or 3’-, 4’-positions, obtaining mono, bis, tri, and tetra-anchored complexes (Figure 11) [82,83]. Substitution with hexylthiophene in 3- or 4-positions was also investigated by impedance spectroscopy (EIS) and open circuit voltage decay (OCVD), revealing that charge recombination with electrolyte solution is largely promoted when compared to the carboxylic-modified one (Figure 10) [84,85]. Efficiencies close to the BD reference were recorded for the tetra-anchored complex **13**, and for the 4’’-thienyl dicarboxy substituted complexes **9**. The symmetric substitution with two hexyltiophene groups was also taken into consideration [86,87].

#### Quaterpyridine Ligand

**Figure 12:**
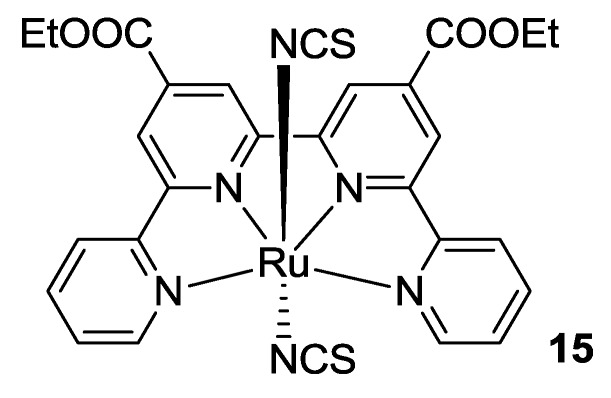
The first qtpy complex applied in DSCs by Renouard *et al.* [90].

Tpy modification included the design of tetrapyridines as tetradentate ligands, that were proposed in order to avoid the geometrical isomerism of bipyridine complexes that leads to *cis* and *trans* conformers, showing different optical properties [88]. In fact, *trans* isomers of bipyridines complexes show better photophysical properties, but they are converted by thermal and photoinduced isomerization to the more stable *cis* isomers that, unfortunately, show worse panchromatic absorption. Tetradentate ligands, owing to their planar structure, coordinate the ruthenium in the plane and only leave apical position available for ancillary ligands, thus avoiding the isomerization and ensuring better solar harvesting features. The first example of a tetradentate ligand for DSCs applications was proposed in 2001 by Renouard *et al.* [89] who synthesized a 6,6’-bis-benzimidazol-2-yl-2,2’-bipyridine and a 2,2’:6’,2’’:6’’,2’’’-quaterpyridine bearing ethyl ester functionalities. The qtpy ligand was then characterized for DSCs applications as a complex with Ruthenium (**15**, Figure 12) [90]. The ester moieties showed poor adsorption on TiO_2_; thus, a further hydrolysis step proved mandatory in order to anchor the dye to the semiconductor surface. Thiocyanate ancillary ligands resulted in blue shifted absorption with respect to chlorine ones due to the stronger σ-acceptor properties of SCN. Remarkable conversion efficiency was recorded, up to 940 nm with 75% IPCE in the plateau region and 18 mA/cm^2^ *J_sc_* (TiO_2_: 12 μm, dye: 0.3 mM ethanol / DMSO 95:5, electrolyte: 0.6 M DMPII, 0.1 M I_2_, 0.5 M t-bupy, 0.1 M LiI in methoxyacetonitrile).

**Figure 13:**
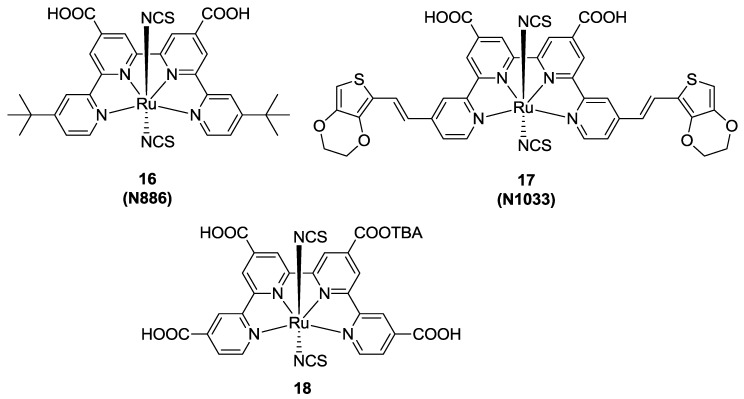
Qtpy complexes investigated by Barolo *et al.* [68,91,92].

A further investigation was reported by Barolo *et al.* [91], in 2006, with the lateral functionalization of the quaterpyridines with *t*-butyl moieties as electron-releasing, bulky groups (**16**, Figure 13). The proposed dye, named **N886**, showed remarkable differences between protonated and non-protonated forms. Wider absorption with respect to N719 was reported, together with a lower molar extinction coefficient and unfavourable alignment of its excited state (as demonstrated by DFT calculations). With the purpose of overcoming these drawbacks, in 2011 the same research group proposed to substitute *t-*butyls with EDOT-vinylene groups, to further extend the π-conjugation (**N1033**, Figure 13) [92]. This complex showed a lower energy gap and a broad IPCE curve having still 33% conversion at 800 nm. The poorer efficiency with respect to **N886** was ascribed to a lower driving force for electron injection, that limits the open circuit potential. The same drawback was also reported for a qtpy substituted with four COOH anchoring moieties (**18**, Figure 13) [68] but its high charge injection and an optimization of the electrolyte composition led to a record efficiency for qtpy Ru-complexes of 6.53% (TiO_2_: 12 + 5 μm, dye: 0.18 mM *t*-butanol / CH_3_CN 1:1 with 10% DMF, electrolyte: 1.0 M dimethylimidazolium iodide, 0.03 M I_2_, 0.1M CDCA, 0.1M GuSCN, 0.23 M LiI in valeronitrile / CH_3_CN 15:85). Co-sensitization with D35, in order to enhance conversion at higher frequencies, was also reported.

### 3.2. Substitution of Ancillary Ligands: Heteroleptic and Cyclometalated Complexes

A further modification on terpyridine complexes involved the substitution of commonly used thiocyanate ligands with other ancillary ligands. The monodentate thiocyanate ligand has the role to tune the spectral and redox properties of the sensitizers acting on the destabilization of the metal t_2_g orbital [93]. By exchanging these ligands with σ-donor groups, it was possible to tune the photochemical properties of the complex, and to minimize the drawbacks associated with these monoanchored ligands. In fact, the possible formation of isomers, owing the bidentate character of the thiocyanate ligand causes a decrease in the synthetic yield [21,78,94]. Moreover the weak Ru-NCS bond itself leads to a decreased stability of the complex and, more importantly, thiocyanate lacks of an effective chromophore that could improve IPCE, particularly at shorter wavelengths. All these features encouraged the engineering of new heteroleptic cyclometalated complexes starting from Black Dye, by exchanging one or more thiocyanate ligands. A drawback affecting this kind of modification is the destabilisation of HOMO orbitals that can lead to a lower driving force in the dye regeneration by the electrolyte.

Strategies for the design of Ru tridentate heterocyclic ligands tailored to tune the properties of the excited state were recently reviewed by Pal *et al.* [95]. Medlycott [96] in 2005 surveyed the strategies for improving the photophysical properties of tridentate ligands commonly considered weaker than bipyridine ones, and Hammarstrom *et al.*, in 2010 [97], investigated the possibility to expand their bite angle. In the following paragraphs we will report an overview of ancillary ligands properly synthesized to tune the photoelectrochemical properties of tpy for applications in DSCs.

#### 3.2.1. Bipyridines

**Figure 14:**
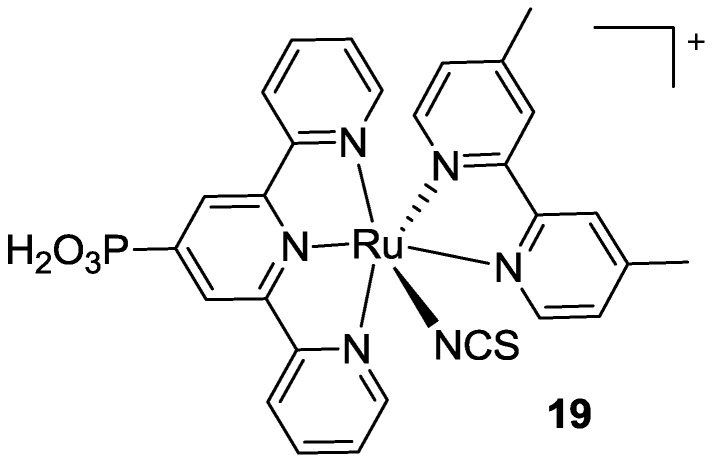
First example of tpy Ru-complex showing a bipyridine instead of two thiocyanates [25].

Ancillary ligand exchange was pioneered in 1997 by Zakeeruddin *et al.* [25] who substituted two of the three thiocyanates with a 4,4’-dimethyl-2,2’-bipyridine. In this case, the tpy ligand was not represented by tctpy, but by a simpler tpy with a phosphonic acid anchoring group (Figure 14).

**Figure 15:**
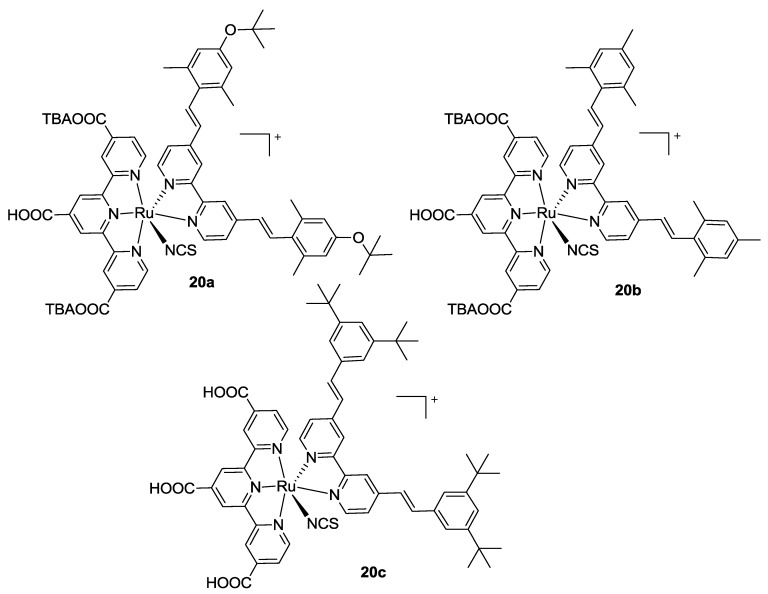
Monothiocyanate complexes proposed by Chandrasekharam [98] (**top**) and Giribabu [99] (**bottom**).

This research topic became of interest again when, in 2011, Chandrasekharam *et al.* [98] proposed to substitute two thiocyanate ancillary ligands with a bipyridine having electron donor styryl moieties in 4,4’- position (**20a,b**, Figure 15). Worse panchromatic behavior was observed with respect to BD, but also better performances in device, owing to an increased molar extinction coefficient in the visible region. A low value of fill factor led to a 3.36% best efficiency, higher with respect to that of BD evaluated in the same conditions (TiO_2_: 9 + 4.8 μm, ethanol solution, Z580 electrolyte: 0.2 M I_2_, 0.5 M GuSCN, 0.5 M N-methylbenzimidazole in [bmim] [I] / 1-ethyl-3-methylimidazolium tetracyanoborate 65:35). Similar bipyridines, slightly modified in the styryl substitution, were also tested by Giribabu *et al.* [99] (**20c**, Figure 15). A more positive oxidation potential with respect to BD under the same conditions has been reported (0.78V *vs.* 0.60V) which was associated with a more negative reduction (−1.30V *vs.* −1.10V) explaining the loss in panchromatic absorption.

**Figure 16:**
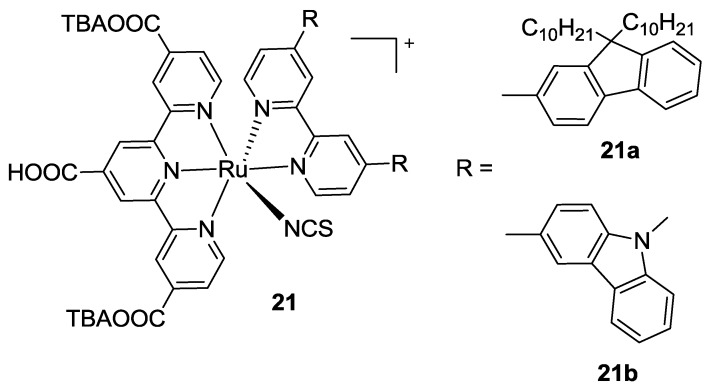
Bipyridine ancillary ligands with fluoren-2-yl or carbazol-3-yl substitutions [100].

Very recently, Koyyada *et al.* [100] reported other bipyridines 4,4’- substituted with fluoren-2-yl (**21a** in Figure 16) or carbazol-3-yl (**21b**) groups, as ancillary ligands. Even if the proposed structures reported good molar extinction coefficients and favourable oxidation and reduction potentials, the overall performances were quite low, mainly due to the poor generated photocurrent that was possibly related to an unfavorable localization of LUMO, far from the anchoring sites on titania.

**Figure 17:**
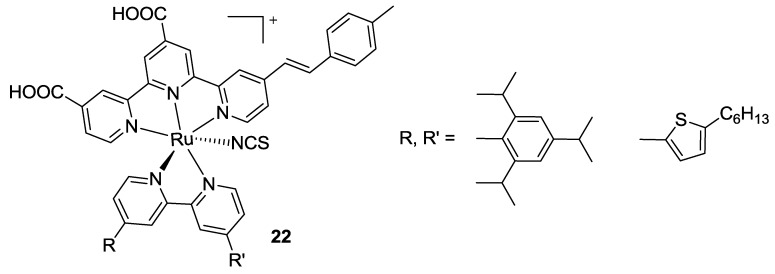
Ancillary ligands modifications of complex **6** [101].

In 2015 Pavan Kumar *et al.* [101] modified complex **6** [77] by substituting two thiocyanates with an asymmetrical bipyridine ligand bearing hexylthiophene and mesityl subtituents on each pyridine ring (**22**, Figure 17).

**Figure 18:**
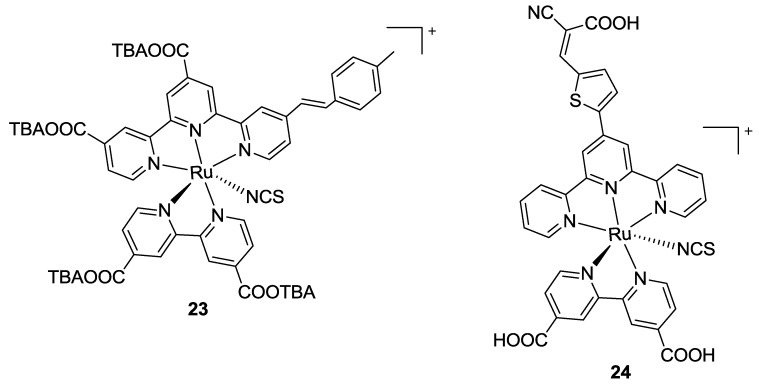
Four (**23**) and three (**24**) anchored complexes by Pavan Kumar [101] and Kanniyambatti [76].

In the same paper, a Ru complex was reported, in which the bipyridine bears two carboxyl substituents. While having four anchoring groups, this complex led to lower efficiencies (**23**, Figure 18). With similar purposes, Kanniyambatti [76] modified complex **5**, achieving a three-anchored sensitizer (**24**, Figure 18) with higher molar extinction coefficient and higher efficiency with respect to both complex **5** and BD tested in the same conditions (*η* = 7.5 vs 6.1%; TiO_2_: 10 + 4 μm, dye: 0.5 mM *t*-butanol / acetonitrile 1:1 with with CDCA 0.5 mM, electrolyte: 0.6 M [bmim][I], 0.03 M I_2_, 0.1 M GuSCN and 0.5 M t-bupy in CH_3_CN / valeronitrile 85:15).

**Figure 19:**
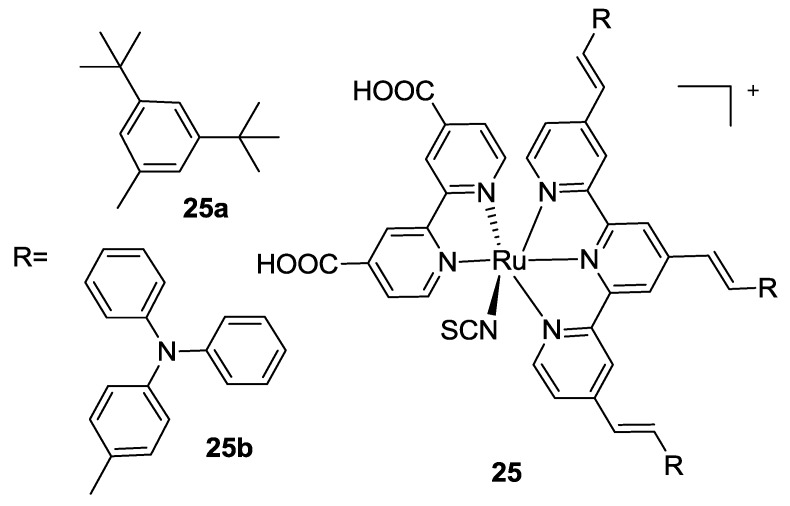
Tpy extended with substituted stiryl moieties by Giribabu [102].

All these modifications were in line with the results from Giribabu, who proposed a Ru complex with 4,4’-dicarboxybipyridine and a tpy ligand bearing the same electron donor in 4,4’,4’’- positions (*t*-butyl or biphenyl amino substituted styryl moieties) (**25a-b**, Figure 19) [102]. In this case, a further enhancement in π-conjugation led to increased molar extinction coefficients and improved performances. Similar complexes that bear donating groups on the terpyridine and electron withdrawing/grafting moieties on a bidentate ligand have been proposed by Mosurkal [103], Erten-Ela [104] and, more recently, by Mongal [105]. In the first case, the anchoring moiety was provided by 4,4’-dicarboxy-2,2’-bipyridine. Mono and dinuclear ruthenium complexes were compared on the device, where the latter one gave better performances. In the second case, the bidentate ligand was represented by a phenantroline substituted with phenyl sulfonic acid moieties in order to graft and sensitize TiO_2_ and ZnO.

#### 3.2.2. Bis-Terpyridine

**Figure 20:**
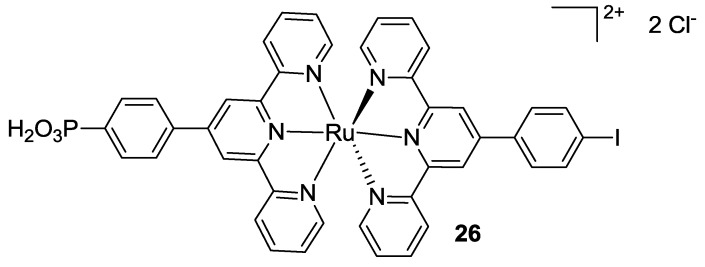
Bis-tpy complex proposed by Stergiopoulos *et al.* [106].

Stergiopoulos *et al.*, in 2005 [106], replaced all the thiocyanates with another terpyridine. In the resulting heteroleptic complex, one tpy was substituted in 4’- with a *p-*iodophenyl moiety and the other one with a *p*-phenylphosphonic acid, in order to allow the grafting to TiO_2_ semiconductor in a solid state device (**26**, Figure 20).

**Figure 21:**
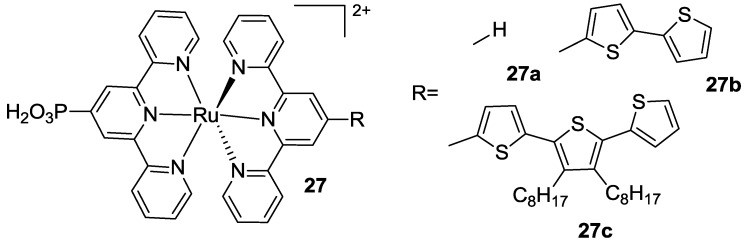
A first series of bis-tpy complexes proposed by Houarner *et al.* [107].

In the same year Houarner *et al.* [107] proposed another bis-tpy complex with a phosphonic acid as the anchoring group on one terpyridine and oligothiophene moieties on the other one, in order to increase the interaction between dye and hole transporting material (**27**, Figure 21). Low performances of this class were attributed to an undesired localisation of the LUMO orbital on thiophenes and, as a consequence, to a difficult charge injection into the TiO_2_. In order to improve the performances, the same group in 2007 introduced an unconjugated bridge between the tpy and the polythiophene moiety [108].

**Figure 22:**
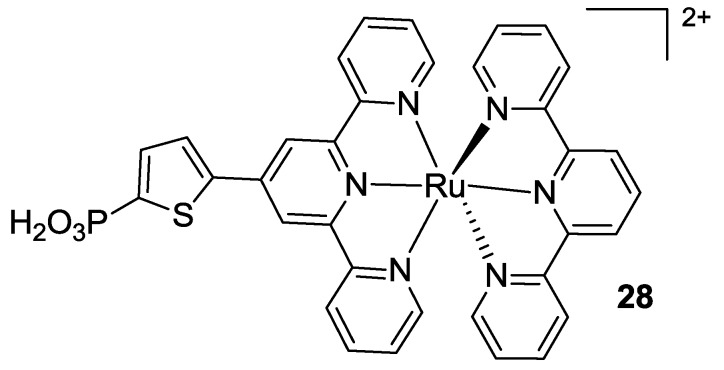
A structural variation of bis-py Ru complex proposed by Houarner *et al.* [109].

Further improvements to the Houarner series were reported in 2007 [109] by introducing a thiophene π-conjugated bridge between the terpyridine and the phosphonate anchoring group, improving the photoconversion efficiency (**28**, Figure 22). The thiophene spacer proved to be an interesting and efficient relay in the molecular design; however, overall low efficiencies were obtained, owing to a lower driving force for charge injection.

Krebs and his research group [110] further investigated bis-tpy Ru complexes using bromophenyl, carboxyphenyl, carboxyl acid [111], and ester moieties in order to compare their anchoring properties. Ester moieties showed weaker absorption to TiO_2_ with respect to carboxylic acid and non-symmetric complexes reported efficiencies three times higher with respect to symmetric ones. The same group [112,113] and Chan [114] studied bis-tpy Ru-complexes in conjugated polymers, and their application to polymeric solar cells [112,113]. Tpy-bearing polyphenylene-vinylene and thienyl-fluorene units were exploited in order to incorporate the resulting Ru complexes in the polymer chains; carboxyl acid functionalization of the bipyridine moieties resulted in improved efficiency. Caramori *et al.* [115], using an heteroleptic thienylterpyridine Ru complex, improved the electron collection efficiency owing to an electrolyte based on the combination of cobalt and iron polypyridine complexes.

**Figure 23:**
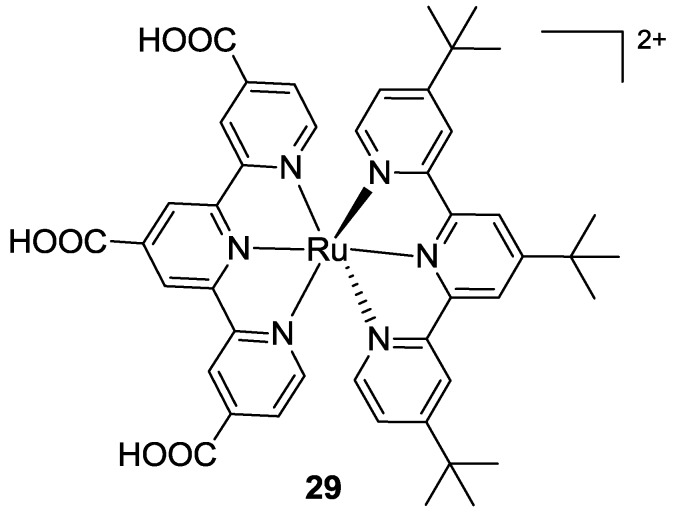
Modification of the BD structure with tris (*t*-butyl) tpy [100].

Very recently, Koyyada [100] replaced all thiocyanates in the BD structure with a tris (*t*-butyl) tpy, thus maintaining tctpy as the anchoring moiety (**29** in Figure 23). The complex showed good optical properties, with a hypsochromic shift in the visible range of the spectrum and a higher molar extinction coefficient respect to BD, but the overall performances were quite low. 

#### 3.2.3. Phenylpyridine and Pyrimidine

**Figure 24:**
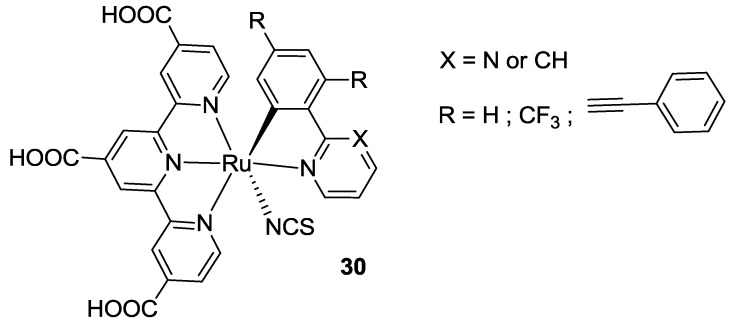
C^N bidentate ligands proposed by Funaki *et al.* for Ru(II)-complexes [116,117,118].

Funaki investigated the possibility to maintain the same terpyridine ligand of Black Dye, tctpy, substituting two thiocyanates with a series of C**^**N bidentate ligands (**30** in Figure 24) [116,117,118]. These complexes were designed in order to utilize ancillary ligands with stronger donor properties with respect to thiocyanates in order to destabilize the t_2_g HOMO orbital, to reduce the band gap and to harness lower energy regions of the solar spectrum. 2-Phenylpyridines as such, and those substituted in 4’ position with a phenyl ethynyl group [118], were used to obtain cyclometalated ruthenium(II) complexes. The wider π-extension allowed to obtain higher molar extinction coefficients and a higher charge injection with an IPCE value of 10% at 900 nm. The main drawback of these complexes was a low oxidation potential that reduced the driving force for dye regeneration. In order to raise the HOMO level and ease the dye regeneration by iodine, the same group [116] extended the C^N ligands series to 2-phenylpyrimidines, substituted on the phenyl ring with trifluoromethyl groups. The CF_3_ group further reduces the electron donor behavior of the ligand and stabilizes the HOMO level. In this way a 10.7% efficiency was obtained, with respect to 10.1% of BD tested in the same conditions (TiO_2_: 25 + 6 μm, dye: 0.4 mM ethanol with 40 mM DCA, electrolyte: 0.6 M DMPII, 0.05 M I_2_, 0.1 M LiI, 0.5 M t-bupy in CH_3_CN). These ligands were further investigated in 2013 [117] by computational studies.

#### 3.2.4. β Diketonate Ligands

**Figure 25:**
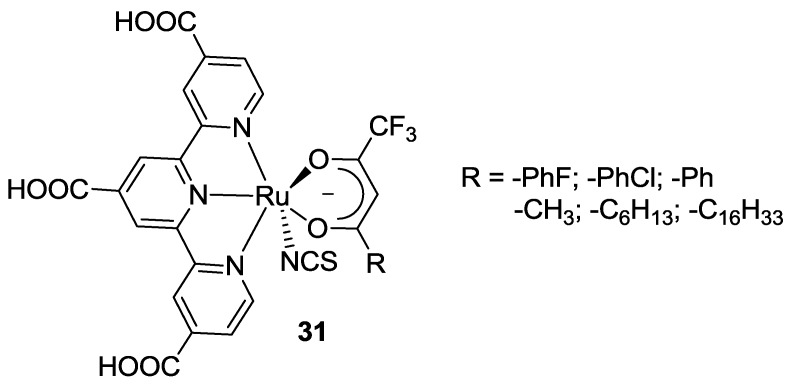
β-diketonates ligands by Islam *et al.* [119,120,121,122,123,124,125].

A series of β-diketonate ligands (**31** in Figure 25) was investigated by Islam *et al.* [119,120,121,122,123,124,125] as ancillary ligands alternative to thiocyanates in the BD structure. The strong σ-donating nature of the negatively-charged oxygen donor atom destabilizes the ground-state energy level of the dye compared to BD, leading to a shift of the MLCT transitions to lower energies. In 2002 [125] a Ru(II) complex with 1,1,1-trifluoropentane-2,4-dionato ligand showed efficient panchromatic sensitization of nanocrystalline TiO_2_ solar cells. Additionally, a longer alkyl chain (using 1,1,1-trifluoroeicosane-2,4-dionato ligand) [122] prevented surface aggregation of the sensitizer and allowed to avoid or reduce the use of chenodeoxycholic acid. The use of longer alkyl chains may protect the TiO_2_ surface, through steric hindrance and hydrophobic effect, preventing the access of electrons to the redox electrolyte, favouring a higher *V_oc_*. On the other hand, the bulky alkyl group may not only facilitate the ordered molecular arrangement on the TiO_2_ surface, but also keep dye molecules far away each other, thus suppressing intermolecular dye interaction and increasing *J_sc_* [126].

In 2006 [123] the same group further modified the β-diketonate ligand with a halogen *p*-chlorophenyl group. Aryl substituents with different electron-donating strength were allowed to control the shift of the low-energy MLCT band and Ru oxidation potential. A very efficient sensitization (*η* = 9.1%; TiO_2_: 20 μm, dye: 0.2 mM CH_3_CN / *t*-butanol 1:1 with 20 mM DCA, electrolyte: 0.6 M DMPII, 0.05 M I_2_, 0.1 M LiI, 0.07 M t-bupy in CH_3_CN), with an IPCE greater than 80% in the whole visible range extending up to 950 nm was obtained. Further substituted β-diketonate ligands were tested in 2011 [119] showing a great potential to tune the photochemical properties.

#### 3.2.5. Pyrazolyl Ligands

**Figure 26:**
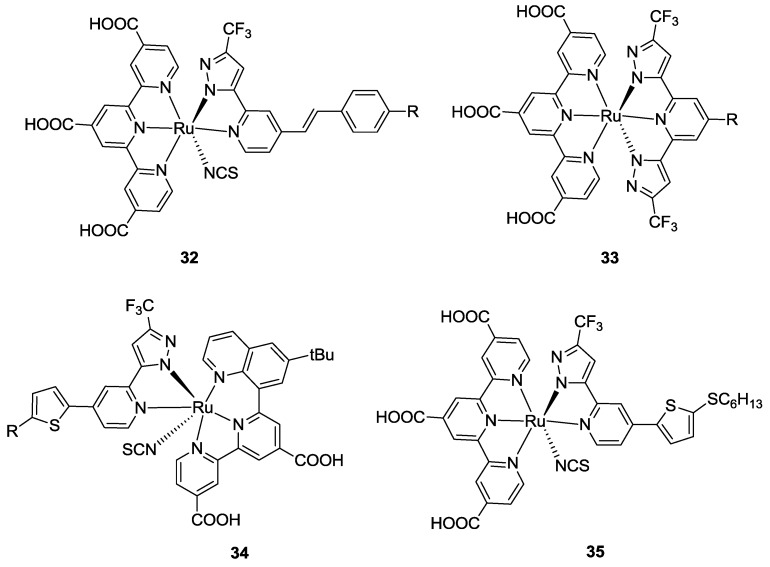
Pyridyl-pyrazolate ligand and quinolyl-bipyridine ligand for Ru(II)-complexes [22,127,128,129].

Novel N^N bidentate ligands, different from the bipyridines, were proposed by Chen *et al.* [127]. A series of 2-(pyrazol-3-yl)pyridine ligands were used as an alternative to thiocyanate in BD and tested in cells (**32**, Figure 26). These dyes overcome the efficiency of BD tested in the same conditions (*η* = 10.05 vs 9.07% ; TiO_2_: 18 + 4 μm, dye: 0.3 mM DMF / *t*-butanol 1:1 with 10 mM DCA, electrolyte: 0.6 M DMPII, 0.1 M I_2_, 0.1 M LiI, 0.5 M t-bupy in CH_3_CN) due to their higher molar extinction coefficients between 400 and 550 nm and their extended absorption up to 850 nm, as a consequence of the HOMO destabilization by the pyrazole. The same group reported, in 2011 [128], a series of tridentate 2,6-bis(3-pyrazolyl)pyridine ligands bearing various substitutions in 4- position (**33**, Figure 26). The reported IPCE spectra showed a worse sensitization in the NIR region with respect to N749 but a better conversion in the visible range which accounts for efficiencies up to 10.7% (TiO_2_: 15 + 5 μm, dye: 0.3 mM ethanol / DMSO 4:1 with 1M CDCA, electrolyte: 0.6 M DMPII, 0.1 M I_2_, 0.5 M t-bupy, 0.1 M LiI in CH_3_CN). The results were explained by the bulky ligand effect, which may allow better packing of the dye molecules on the TiO_2_ surface and prevent interfacial charge recombination. On the other hand, the contribution of the pyridine in the ligand, which is neutral with respect to the negatively charged thiocyanates, might allow the negative dipole moment to be localized closer to the surface, thus affording a higher *V_oc_*. Further investigations on these complexes were carried out by replacing the tctpy with a dicarboxytpy ligand substituted in the 5- or 6- position of a terminal pyridyl unit with π-conjugated thiophene pendant chains, obtaining good stability and performances with respect to BD [129]. More recently, the terminal pyridyl unit of the tctpy was replaced with variously substituted quinolines (**34**, Figure 26) reaching good performances (*η* = 10.19%; TiO_2_: 15 + 7 μm, dye: 0.3 mM ethanol / *t*-butanol 1:1 with 0.6 mM of tetra-butylammonium deoxycholate, electrolyte: 0.6 M DMPII, 0.05 M LiI, 0.05 M I_2_, 0.5 M t-bupy in CH_3_CN) [130]. In this new family of complexes, electron-donating-bulky *t*-butyl substituents on quinoline gave better performances with respect to the electron-withdrawing COOH group. With the *t*-butyl group, in fact, a blueshift for transitions at lower energies was reported together with a hyperchromic effect that improved IPCE and *J_sc_*. Further modifications to the bidentate ancillary ligands led to a best result efficiency (*η* = 11.16%; with the addition of 1mM DCA as co-adsorbate to the dye solution and electrolyte: 0.1 M LiI and 0.1 M GuNCS, 0.5 M t-bupy in CH_3_CN) [22] when tctpy and hexylthiothienyl-substituted pyrazolyl-pyridine were used to complex ruthenium (II) (**35**, Figure 25). This complex showed worse conversion in the NIR spectral region but improved IPCE in the visible one with respect to BD, thus determining a better efficiency in the same conditions.

Recently, Chang *et al.* [131] reported pyrazolyl-pyridine ancillary ligands bearing a series of donor groups in which the simplest substituents (such as *t*-butyl group) leads to better efficiency with respect to the triphenylamino and benzothiadiazolylgroups.

#### 3.2.6. Phenyl Bipyridines

**Figure 27:**
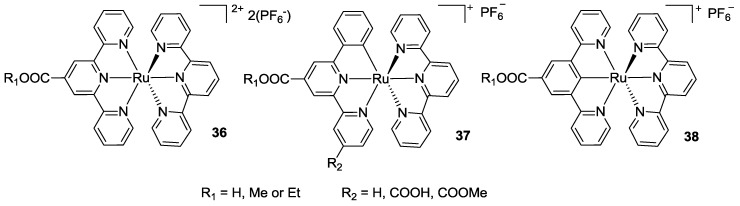
Tpy and phenyl-bipyridines complexes investigated by Wadman *et al.* [132].

In 2007 Wadman *et al.* [132] compared a bis-tpy Ru complex bearing one carboxyl group in the 4- position, with two structurally homolog complexes in which the tpy was replaced by 6-phenylbipyridines, with one or two carboxyl groups (**36** and **37**, Figure 27). The N^N’^C Ru(II)-complex with two anchoring groups showed performances similar to N719. Thus, in 2010 [133], the same group further extendend the series, including N^C^N’ ligands based on 3,5-bis(2-pyridyl)benzoic acid (**38**, Figure 27). 

**Figure 28:**
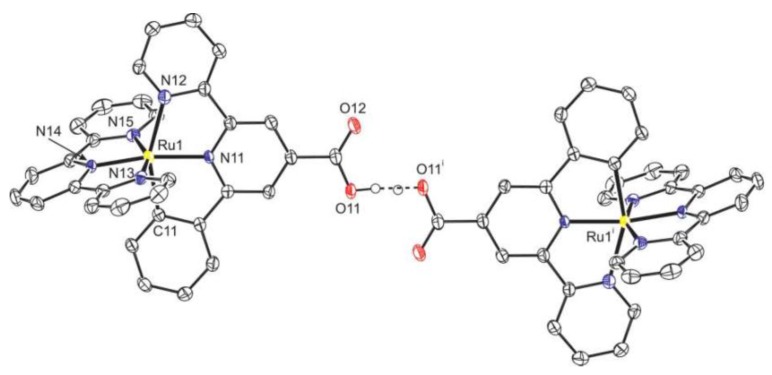
Crystal structure of complex **37** in form of its dimer [132] (Adapted from Ref 131 with permission of The Royal Society of Chemistry).

X-ray structural determination on the mono carboxyl complex (**37**, R_1_ = H, R_2_ = COOH) showed a distorted octahedral coordination, with the cyclometalated ligand perpendicular to the terpyridine and elongation of the nitrogen to Ru bond opposite to the C-Ru bond. In the solid state, the complex forms dimers via hydrogen bonds between the carboxyl functions (Figure 28).

N^N’^C cyclometalated compounds showed better sensitization properties respect to the bis-tpy complexes; while the lower efficiencies of the N^C^N' complexes were ascribed to a LUMO localization which prevented an efficient electron injection into the TiO_2_ conduction band. The replacement of a coordinative Ru-N bond with a covalent carbon-ruthenium bond led to a redshift and to a broadening in the optical absorption of the corresponding ruthenium complex. Functionalization on the N^C^N’ ligand with another tpy resulted in the synthesis of dinuclear Ru(II)-complexes [134].

**Figure 29:**
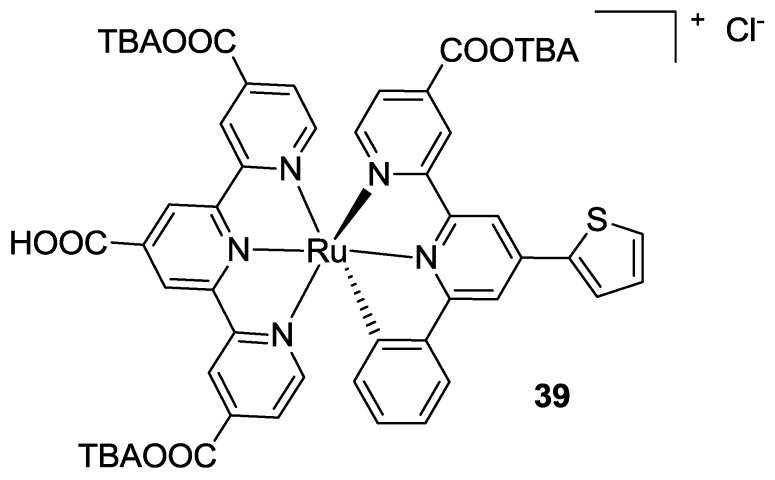
Bis-tpy-based Ru(II) complex proposed by Kisserwan *et al.* [135].

Kisserwan *et al.* [135] further engineered the 6-phenyl-2,2’-bipyridyl (C^N^N’) ligand with a thiophene and carboxylic acid moieties in the 4- and 4’- positions of the bipyridine moiety (**39**, Figure 29). The thienyl group was chosen with the purpose of increasing the molar extinction coefficient, while COOH had the aim to further strengthen the coupling with TiO_2_. With respect to Wadman’s works, tctpy was used instead of tpy. The work focused more on electrolyte composition than on sensitizer design, providing better performances when CuI was used as an additive. The same group in 2012 [57] extendend the investigation on the 6-phenyl-2,2’-bipyridyl (C^N^N’) ligand, studying the influence of either donor or acceptor substituents on the phenyl and the presence of COOH on the bipyridine. When the thienyl group was replaced by COOH, lower efficiencies were observed, attributed to a less efficient electron injection. The best sensitizer was also studied for its long-term stability, showing better results when compared to N719.

**Figure 30:**
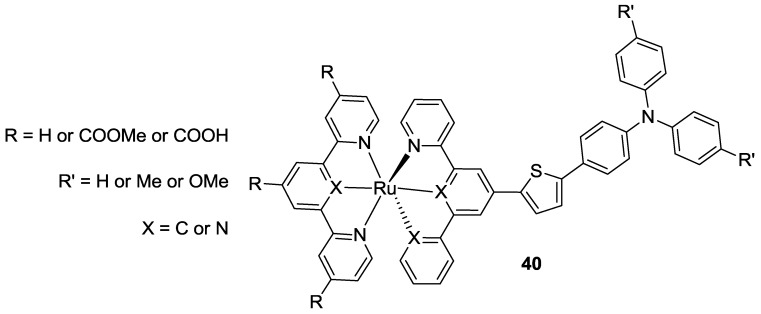
Robson *et al.* [136] series of bis-tridentated ruthenium complexes bearing triphenyl amino groups.

In 2011, Robson *et al.* [136] published an extensive study in which a series of asymmetric bis-tridentated ruthenium complexes was synthesized, whose ligands ranged from terpyridine (N^N’^N’’) to phenyl-bipyridine (C^N^N’) and di-(2-pyridyl)-benzene (N^C^N’), bearing anchoring electron-withdrawing groups on one ligand and, on the other, a thienyl-triphenylamino group as donor counterpart (**40**, Figure 30). A thorough investigation of the photophysical and electrochemical properties was pursued in order to understand the role of the organometallic bond and terminal substituents and to tune the energetic levels. Broad absorption spectra were generated in Ru(II) complexes containing an organometallic bond because of the electronic dissymmetry about the octahedral Ru(II) center. The intensity of the spectra in the visible region was enhanced when the organometallic bond was orthogonal to the principal axis (*i.e.,* C^N^N’ ligand). When the anchoring ligand is represented by a N^C^N’ tridentate combination, the LUMO is placed remotely from TiO_2_, and this prevents an efficient charge injection. On the other hand, if the organometallic bond is placed on the donor ligand, HOMO level can be localized either on the triphenyl amino moiety or on Ru(II), maximizing light harvesting in the visible region; while, at the same time, the LUMO on the anchoring ligand ensures an efficient electron transfer towards the semiconductor surface. The highest recorded efficiency reached 8.02% (TiO_2_: 15 + 4.5 μm, dye: 0.3 mM ethanol, Z1137 electrolyte: 1.0 M 1,3-dimethylimidazolium iodide, 60 mM I_2_, 0.5 M t-bupy, 0.05 M NaI, 0.1 M GuNCS in CH_3_CN / valeronitrile 85:15).

#### 3.2.7. Dipyrazinyl-Pyridine

**Figure 31:**
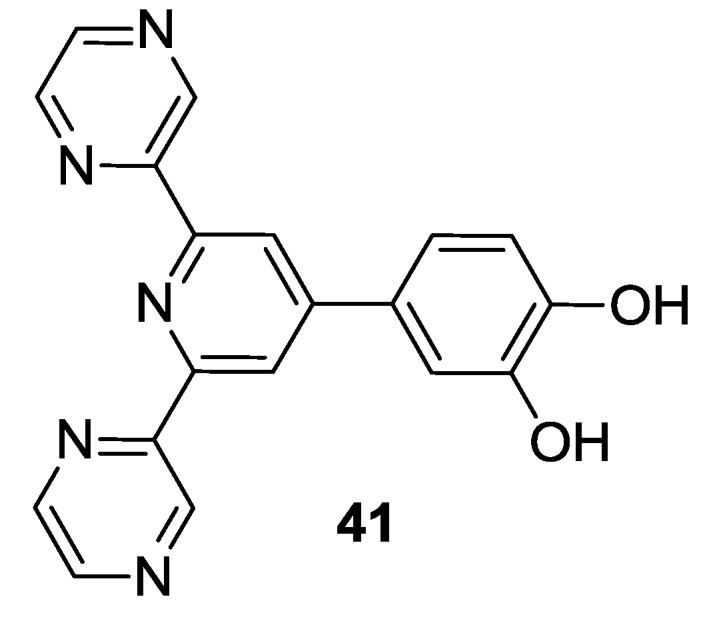
Example of dipyrazinyl-pyridine ligand [135].

Another series of bis-tridentate complexes was reported in 2007 by Al-mutlaq *et al.* [137] using dipyrazinyl-pyridine ligands with different substituents on 4’- position, and cathecol moieties as grafting groups (**41**, Figure 31). In comparison to homolog complexes with terpyridine, dipyrazinyl-pyridine led to higher oxidation potential. Exchanging SCN improved HOMO and LUMO while substituting tpy with dipyrazinyl-pyridine lowered these values.

Sepehrifard *et al.* [138,139] investigated a series of homoleptic bis-tridentate ruthenium complexes, employing both tpy and dipyrazinyl-pyridine ligands. The poorer performances of the latter ones were attributed to lower LUMO levels and weaker bonding to TiO_2_. The best results were obtained with terpyridine ligands bearing COOH grafting groups (1.53% efficiency) while the use of dipyrazinyl-pyridine ligands, ester groups or the introduction of a phenylene spacer between the pyridine and the anchoring group all resulted in lower efficiencies.

#### 3.2.8. Triazolate

**Figure 32:**
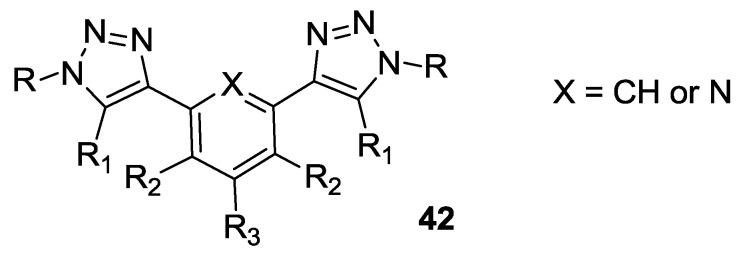
Triazolate ligand studied by Schulze *et al.* [140,141].

Schulze *et al.* investigated triazolate as chelating moiety in a series of N^C^N’ cyclometalated ligands [140] and N^N’^N’’ ligands [141]. 1,3-Di(4-triazolyl)benzene and 2,5-di(4-triazolyl)pyridine were used in association with tctpy as the grafting moiety (**42**, Figure 32). In the case of the N^C^N’ ligand, the substitution with electron-withdrawing groups such as F or NO_2_ stabilizes the HOMO energy level providing blueshift and loss in charge injection, while hydrophobic alkyl chains are expected to be beneficial for the long-term stability. The relatively low efficiency obtained as the best result (*η* = 4.9%; TiO_2_: 12 + 3 μm, dye: 0.25 mM methanol, electrolyte: 0.6 M 1,3-dimethylimidazolium iodide, 0.06 M I_2_, 0.1 M LiI, 0.5 M t-bupy, 0.1 M GuSCN in CH_3_CN) in the case of the N^N’^N’’ ligand with respect to N749 (6.1% in the same conditions, dipping solution in ethanol) was explained by loss in panchromatic absorption.

#### 3.2.9. Other Ligands

**Figure 33:**
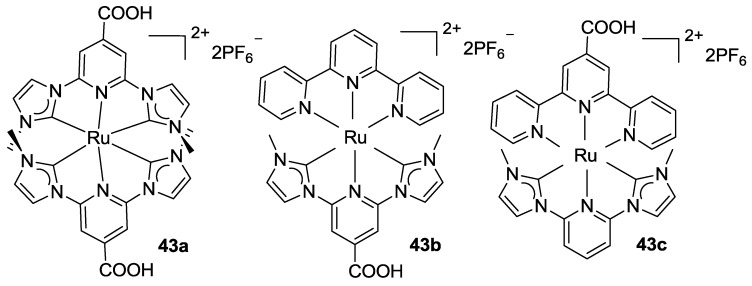
Ru(II) complexes proposed by Park *et al.* [142].

C^N^C’ ligands have been tested by Park *et al.* [142] in a series of bis-tridentate ruthenium complexes, exploiting N-heterocyclic carbenes such as 2,6-bis-(3-methylimidazolium-1-yl)pyridine (**43a-c**, Figure 33). 

**Figure 34:**
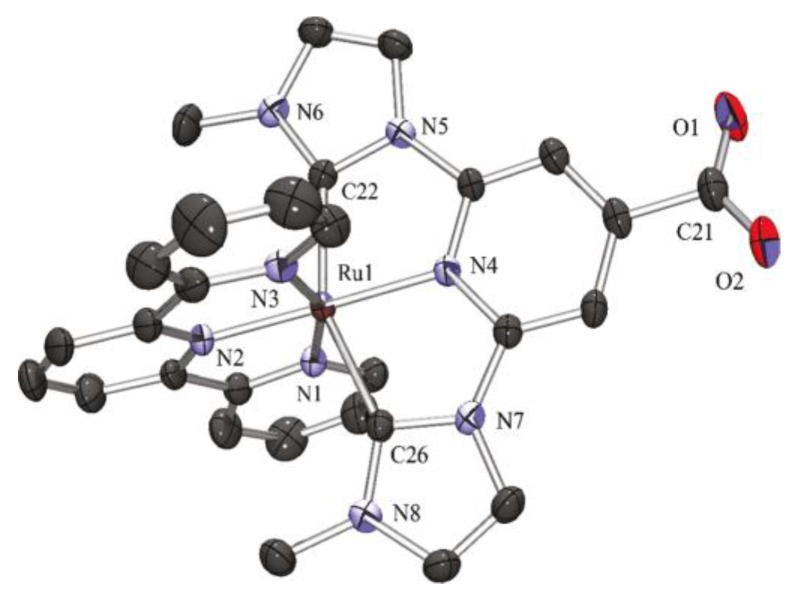
ORTEP drawing of complex **43b** [142] (Reprinted with permission from Park, H.-J.; Kim, K. H.; Choi, S. Y.; Kim, H.-M.; Lee, W. I.; Kang, Y. K.; Chung, Y. K. Unsymmetric Ru(II) Complexes with N-Heterocyclic Carbene and/or Terpyridine Ligands: Synthesis, Characterization, Ground- and Excited-State Electronic Structures and Their Application for DSSC Sensitizers. Inorg. Chem. 2010, 49, 7340–7352. Copyright 2010 American Chemical Society).

X-ray crystal structure of **43b** shows a typical geometry with both ligands coordinated in a meridional fashion; bond distances between Ru and the coordinated N or C are similar and the carboxyl function is deprotonated (Figure 34). Overall efficiencies were far from N719 tested in the same conditions, a result that was mainly attributed to low charge injection.

Bonacin *et al.* [143] proposed a complex of Ru(II) with carboxyphenyl tpy, thiocyanate, and 8-hydroxy quinoline in order to host a carboxymethyl cyclodextrin anchored to TiO_2_. Even if poor results were reported (ascribed to high HOMO potential and low regeneration), the host-guest interaction of the dye with the cyclodextrin increased the performances by preventing dye aggregation and limiting the dark current. 

**Figure 35:**
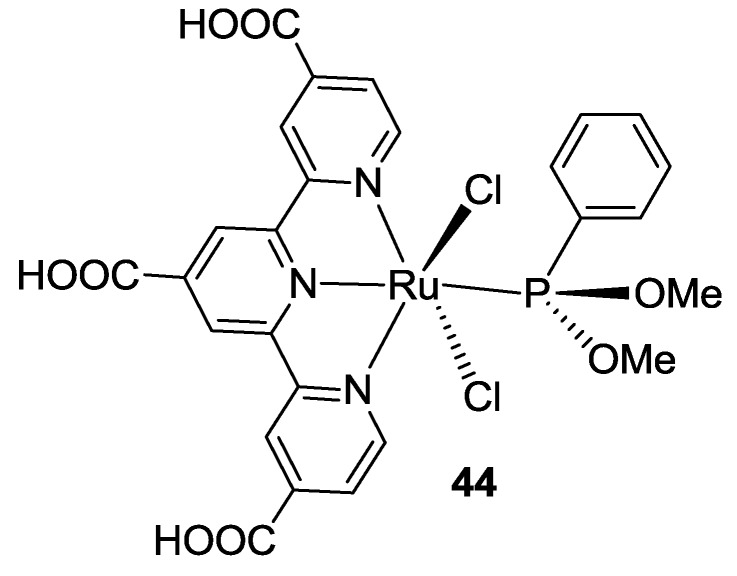
Phosphine-coordinated Ru(II) sensitizer by Kinoshita *et al.* [144].

Kinoshita *et al.* [144,145] spent efforts in order to further extend the absorption of BD. In conventional Ru(II) complexes, short-lived ^1^MLCT states immediately relax to long-lived ^3^MLCT states through intersystem crossing. The spin-forbidden singlet-to-triplet transition from HOMO to ^3^MLCT has been observed for a phosphine-coordinated Ru(II) sensitizer (**44** in Figure 35), providing light conversion up to 1000 nm and unprecedented charge injection (26.8 mA/cm^-2^). Unfortunately no evidence about long-term stability of this complex was reported.

**Figure 36:**
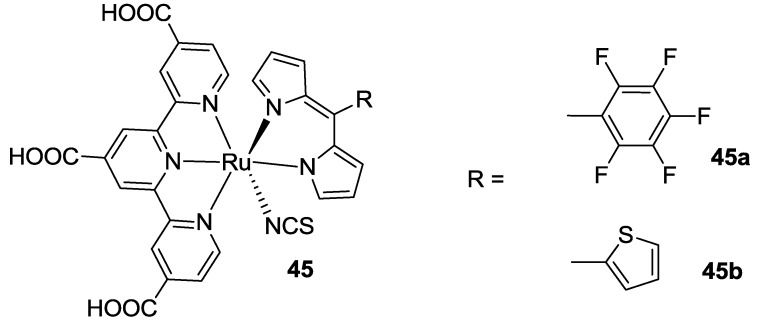
2,2’-Dipyrromethane by Li *et al.* [146].

Recently, Li used 2,2’-dipyrromethanes as N^N’ bidentate ligand in order to substitute thiocyanates in the BD structure. The dipyrromethanes having 5-pentafluorophenyl and 2-thienyl substituents gave IPCE curves showing a sensitization up to 950 nm (**45**, Figure 36) [146].

**Figure 37:**
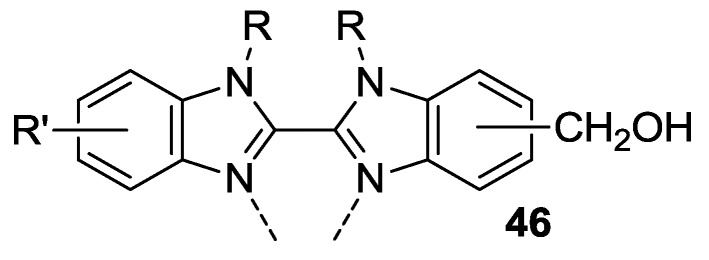
Benzimidazole ligand tested by Swetha *et al.* [147].

A bidentate benzimidazole was tested by Swetha *et al.* [147] as ancillary ligand in a Ru complex with tctpy, showing blueshifted absorption and a higher molecular extinction coefficient in the high energy region of the solar spectrum with respect to N749, which accounted for a better IPCE in the 400–640 nm range and a 6.07% efficiency (**46**, Figure 37; dye: 0.3 mM CH_3_CN / *n*-butanol 1:1 with 20 mM DCA, electrolyte: 0.5 M DMPII, 0.05 M I_2_, 0.1 M LiI CH_3_CN / butanol 1:1).

### 3.3. Exchange of Metal Center

Terpyridine complexes with other metals were reported by Bignozzi’s group, who complexed osmium with tctpy, various bipyridines and pyridylquinoline [148,149,150]. The idea was to further broaden absorption spectra thanks to Os(II) complexes characterized by high spin-orbit coupling constant that allows the direct population of low energy, spin-forbidden, ^3^MLCT states. No significant differences in IPCE values were found in the case of the various Os complexes showing values up to 50% at 900 nm and 70% in the visible region. A better stability was ascribed to Os complexes with respect to the Ru case, even though these complexes showed lower light conversion.

**Figure 38:**
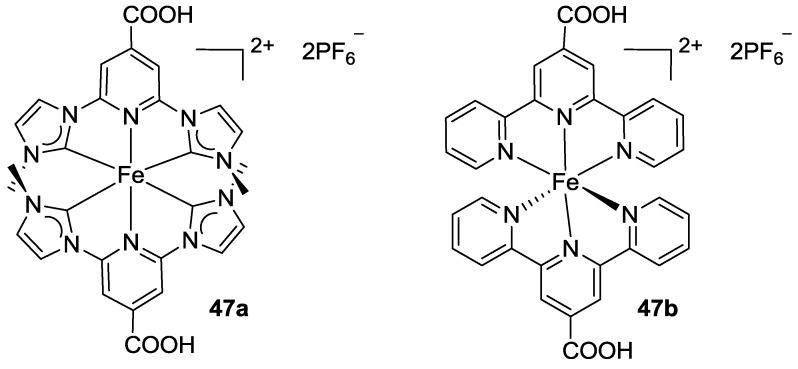
Iron complexes reported by Duchanois [152].

Lapides and co-workers, in 2013 [151], tested another element of the eighth group, iron, using terpyridines as ligands in a supramolecular structure with Ru, as a multicomponent film deposed on TiO_2_. An improved stability of the ruthenium dye was reported, even if these structures have not been tested on DSCs devices. More recently, Duchanois [152] reported a homoleptic iron complex bearing tridentate bis-carbene (C^N^C’) ligands for sensitization of TiO_2_ photoanodes (homologous to the Ru complex **43a**), and compared it with a bis-tpy iron complex (**47a,b**, Figure 38). A considerable stabilization of ^3^MLCT state was obtained for the cyclometalated complex, but still low performances were recorded with respect to the reference sensitizers.

Since platinum(II) complexes usually display an intense charge-transfer absorption band in the visible region, Kwok *et al.* [153], in 2010, proposed a complex of platinum with tctpy and various alkynyl ancillary ligands, reaching up to 3.6% efficiency.

Shinpuku *et al.* [154] synthesized a series of new complexes of iridium with tpy and biphenylpyridine. Cyclometalated iridium complexes were commonly exploited in light source devices as OLEDs and showed narrower absorption spectra respect to Ruthenium ones due to more energetic MLCT transition. A shorter portion of the solar spectrum was harnessed and a lower *J_sc_* was detected, nevertheless a 2.16% efficiency and long lived excited-state lifetime were reported.

The interest for d^10^ metal ions complexes such as Zn-porphyrines has grown in photonic applications, ranging from OLEDs and LECs to DSCs technologies. Bozic-Weber *et al.* [155,156,157] synthesized bis-tpy Zn heteroleptic complexes for TiO_2_ sensitization. Terpyridines substituted with various anchoring and triphenylamino moieties extended with benzothiadiazole-diphenylamino units gave efficiencies between 0.5% and 1%. Housecroft *et al.* reviewed sensitizers made of Earth-abundant metals, concerning copper [158] and other d-block metals [159].

## 4. p-Type

Tpy complexes have been investigated also for the sensitization of p-type semiconductors. In p-type DSCs, the rules for sensitizers design are inverted with respect to classical n-type DSC cells. In fact, in these devices, the excited dye has to inject holes from HOMO to the conduction band of a p-semiconductor [160].

**Figure 39:**
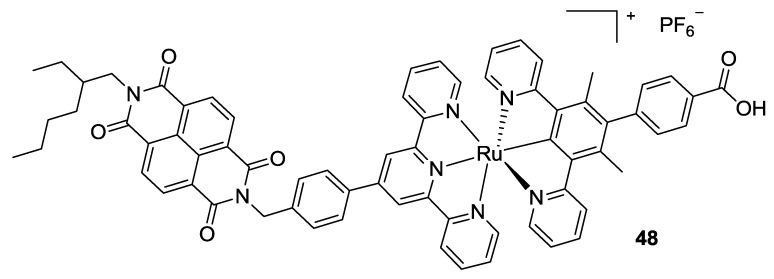
NDI-Tpy proposed by Ji *et al.* [161].

Ji *et al.*, in 2013 [161], proposed a cyclometalated (N^C^N’)-(N^N’^N’’) Ru[II] chromophore to sensitize NiO (**48**, Figure 39). The N^C^N’ ligand was employed as anchoring moiety, while the tpy ligand was functionalized in the 4’ position with a substituted naphthalenediimide (NDI) in order to withdraw electrons from the NiO surface. This dye was studied by femtosecond transient absorption spectroscopy, and results showed a slower charge recombination in the NDI-substituted complex. Perylene imides have been recently used by Sariola-Leikas *et al.* [162] as bridge groups to obtain supramolecular structures for TiO_2_ sensitization in solid state devices.

**Figure 40:**
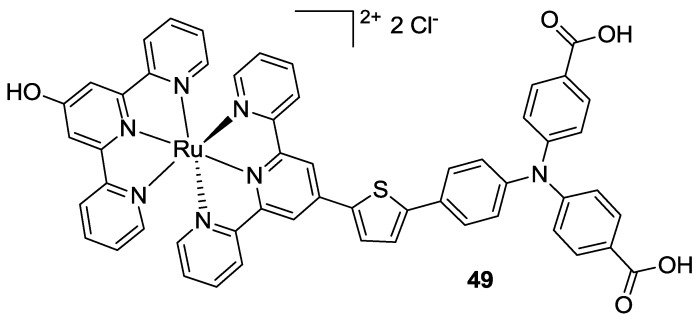
“K1” structure proposed by Wood *et al.* [164].

In 2014 both Constable [163] and Wood [164] proposed heteroleptic tpy complexes for sensitization of p-type semiconductors. The latter used a triphenyl amino moiety as anchoring donor group to increase the hole injection achieving efficiencies in pDSCs between 0.07 and 0.09 (**49**, Figure 40). Both bis-tpy and phenylbipy-tpy complexes were investigated showing better performances with iodine electrolyte with respect to the Co-based one, which was ascribed to high charge recombination with NiO.

## 5. Co-Sensitization

**Figure 41:**
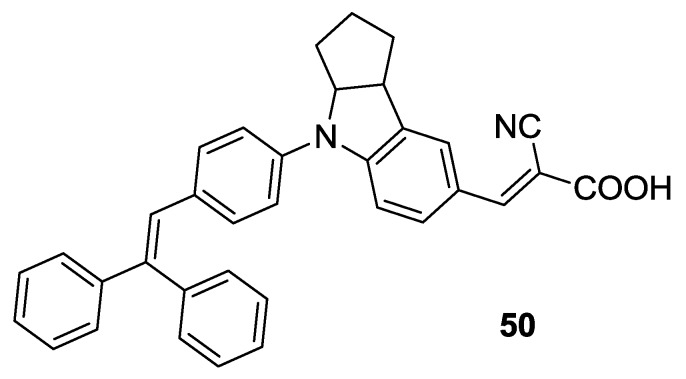
D131 structure used in cosensitization [165,166].

The Black Dye has also been used in cocktail with other sensitizers characterized by higher molar extinction coefficient in the high energy regions of the spectrum, in order to increase the IPCE at lower wavelengths. Ogura *et al.* [165] used BD in combination with the push-pull indoline dye D131 (**50**, Figure 41), reaching a conversion efficiency of 11.0% (working electrode was made with different layers of TiO_2_ mixtures with increasing amounts of polystyrene; 0.19 mM D131 and 0.56 mM BD in CH_3_CN / *t*-butanol 1:1, electrolyte: 0.15 M NaI, 0.075 M I_2_, 1.4 M DMPII, CH_3_CN / methoxyacetonitrile 9:1). Ozawa *et al.* [166] optimized this system using 20 mM chenodeoxycholic acid achieving a 11.6% efficiency with a TiO_2_ film with 45 μm thickness (0.14 mM D131 and 0.2 mM BD in 1-propanol, electrolyte: 0.05 M I_2_, 0.1 M LiI, 0.6 M DMPII, 0.3 M t-bupy in CH_3_CN).

Sharma [167] proposed the cosensitization of a modified BD complex with a Zn porphyrin, with a recorded efficiency of 8.15%. Bahreman [168] synthesized a Ru complex in which a tpy was covalently bound to rhodamine B through an ethanolamine spacer, thus pursuing an energy transfer by “reverse“ FRET.

## 6. Summary and Outlook

The literature offers multiple choices in order to tune the photoelectrochemical properties of terpyridine-based complexes such as the Black Dye, ranging from the modification of the donor and acceptor ligands to the exchange of the metal center with other cations. The increase of the molar extinction coefficient has been commonly pursued by extending the π-conjugation on the ligands. Different anchoring moieties were compared, among which COOH turned out as one of the most effective groups. Isothiocyanate was often substituted by different ancillary ligands in order to improve long-term stability and the synthetic yield of complexation; bidentate and tridentate ligands that exploit coordination through N or C atoms have been tested in order to achieve a better sensitization. Tetradentate ligands have been used in order to further enlarge the spectral absorption properties.

Few outlines can be depicted in this scenario for the design of future complexes: (1) better stability can be achieved avoiding the use of monodentate SCN ancillary ligands; (2) better performances are offered in the case of heteroleptic complexes (the homoleptic ones have an unfavourable symmetric charge distribution); (3) hydrophobic substitutions on the ligands are able to reduce the electron recombination; (4) a better coupling between the complex and semiconductor can be achieved when COOH moieties are used as attaching groups. Overall, a wise approach is requested in order to tune the energy levels far enough to reach panchromatic absorption, but not too much in order not to exceed the limit for a good regeneration rate by the electrolyte and a good electron injection driving force. Furthermore, the use of tpy complexes nowadays goes beyond the traditional role as sensitizers. Cobalt complexes have been reported as redox mediators, by exploiting the interaction of the EDOT-substituted complex with a PEDOT-covered counter-electrode (PEDOT: poly(3,4-ethylenedioxythiophene) [169]. By finely tuning the single DSC components and their interaction, a further increase of DSC performances will be possible.

## Abbreviations

The following abbreviations are used in this manuscript:

BD or N479: Black dye
[bmim][I]: 1-butyl-3-methyl imidazolium iodide
CDCA: Chenodeoxycholic acid
DSCs: Dye sensitized Solar Cells
DCA: Deoxycholic acid
DMPII: 1,2-dimethyl-3-propylimidazolium iodide
EDOT: 3,4-ethylenedioxythiophene
EIS: Impedance spectroscopy
FRET: Förster Resonance Energy Transfer
GuNCS: guanidinium thiocyanate
IPCE: Incident photon to current efficiency
*J_sc_*: Short circuit current
LEC: Light-emitting Electrochemical Cell
MLCT: Metal to ligand charge transfer
NDI: Naphthalenediimide
OCVD: Open Circuit Voltage Decay
OLED: Organic Light Emitting Diode
PEDOT: Poly(3,4-ethylenedioxythiophene)
qtpy: 2,2':6',2":6",2"'-Quaterpyridine
SCN: Thiocyanate
TBA: Tetrabutylammonium
t-bupy: (t-butylpyridine)
tctpy: 4,4’,4’’-Tricarboxyl-2,2’:6’,2’’-terpyridine
TEA: Tetraethylammonium
tpy: 2,2’:6’,2’’Terpyridine
*V_oc_*: Open circuit voltage
